# Supplementary material for: Inhibition of Expression of the Circadian Clock Gene Cryptochrome 1 Causes Abnormal Glucometabolic and Cell Growth in Bombyx mori Cells
Source: Int J Mol Sci. 2023 Mar 12;24(6):5435. doi: 10.3390/ijms24065435 (PMC10056408; doi:10.3390/ijms24065435)
Supplement: Supplementary file 1 [file ijms-24-05435-s001.zip › ijms-2248137-supplementary.pdf]

Supplementary Information

Supplementary Figures and legends

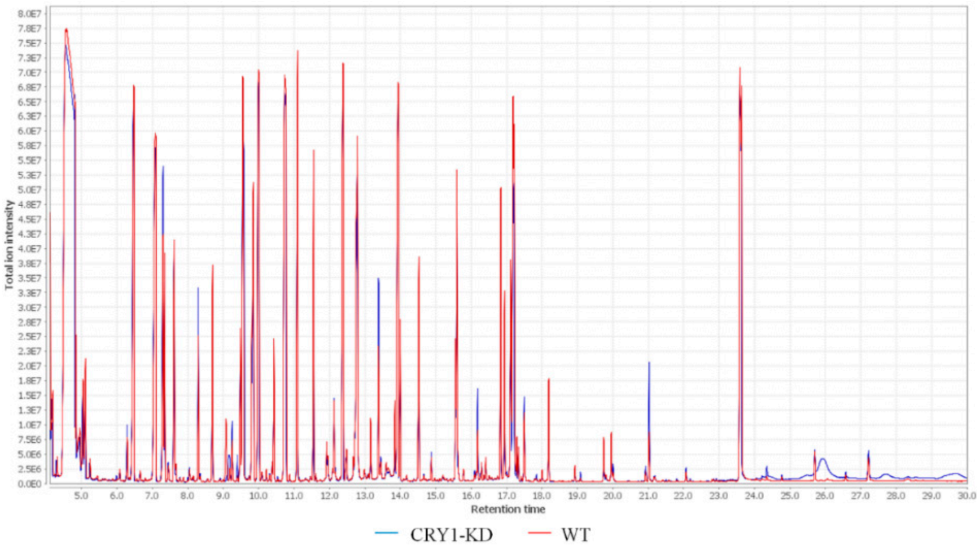

Figure S1. Total ion current GC-MS chromatogram. GC conditions: split injection with a split ratio of 20:1 and an injection volume 1 μL. The inlet temperature was 280 °C; the ion source temperature was 250 °C; and interface temperature 150 °C. The initial temperature of programmed heating was 80 °C and maintained for 2 min; the temperature was then raised to 300 °C at a rate of 10 °C /min and maintained for 5 min. The total operating time was 30 min; the carrier gas was helium; and the carrier gas flow rate was 1 mL/min. MS conditions: an electrospray ionization (ESI) source, full scan mode, and electron energy of 70 eV were used; with a quadrupole scan range of m/z 35 ~ 780.

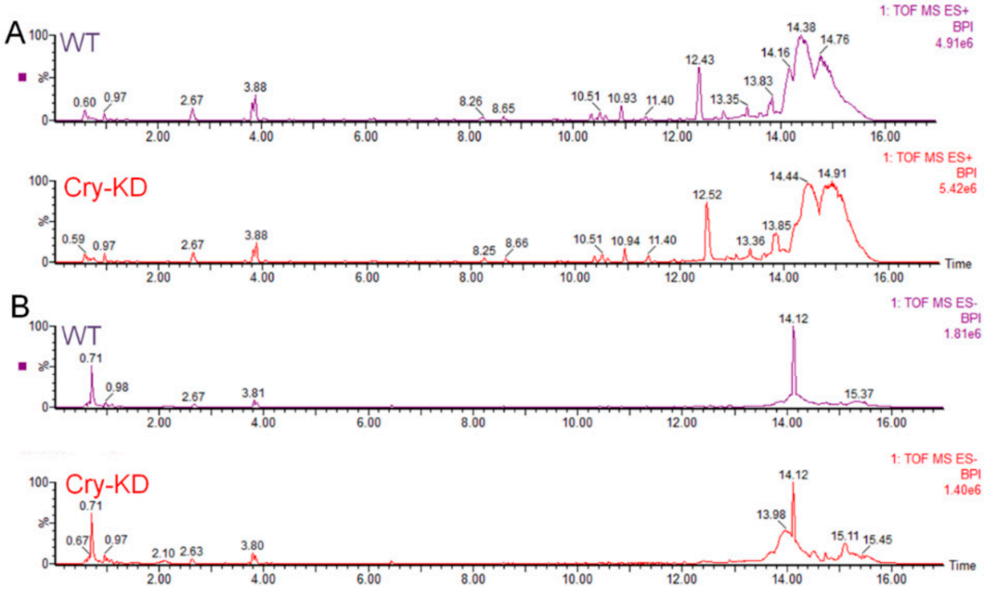

Figure S2. Total ion current LC-MS chromatograms. **(A)** Positive ion mode. **(B)** Negative ion mode. Mobile phase A/B was ultrapure water/acetonitrile, which consisted of 0. 1% (V/V) formic acid with a flow rate of 0.3 mL/min. A Linear gradient elution was used with an injection volume 2 μL and a column temperature of 40 °C; the autosampler temperature was maintained at 4 °C. Leucine enkephalin as lock and spray (0.4 ng/L, 0.1% formic acid CAN/H_2O 50/50). MS conditions: ESI source and positive and negative ionization modes were

used. The source temperature was 120 °C; the desolvation temperature was 350 °C; the desolvation gas flow was 800 L/ h; and the cone gas flow was 50 L/ h. The capillary ionization voltages of positive and negative ion mode were 3.0 kV and 2.8 KV, respectively, with a sampling cone of 30 eV; and extraction cone of 4 eV; and a quadrupole scan range of m/z 50 ~ 1000.

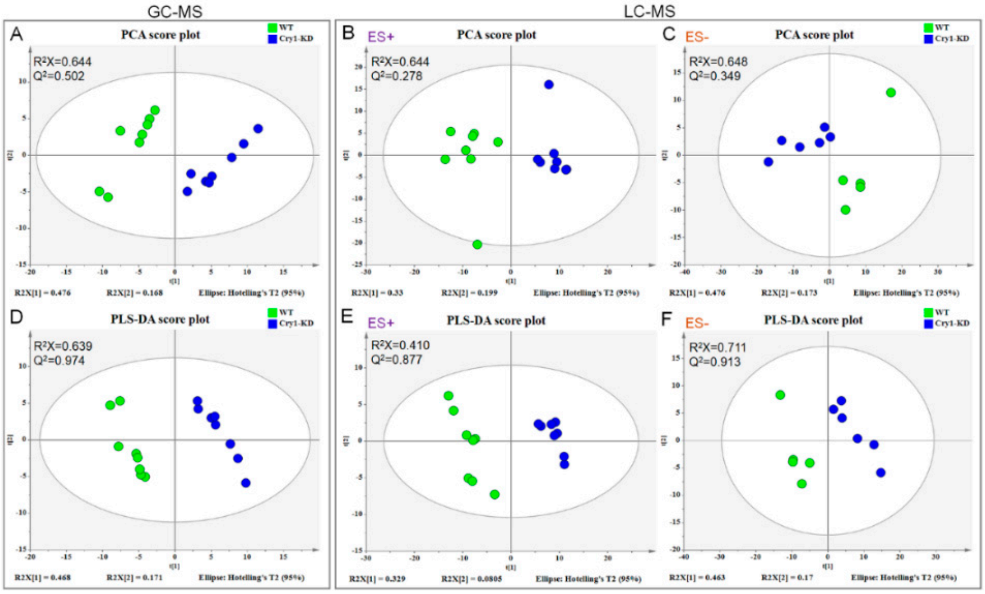

Figure S3. PCA and PLS-DA analysis of metabolites between WT and Cry1-KD samples were compared. **(A & D)** PCA and PLS-DA analysis of GC-MS data. **(B & E)** PCA and PLS-DA analysis of GC-MS data in positive ion mode. **(C & F)** PCA and PLS-DA analysis of GC-MS data in negative ion mode. R^2X and Q^2, which represent the variables that can be explained by the model and the predictability of the model, respectively, are used to evaluate the model's effectiveness.

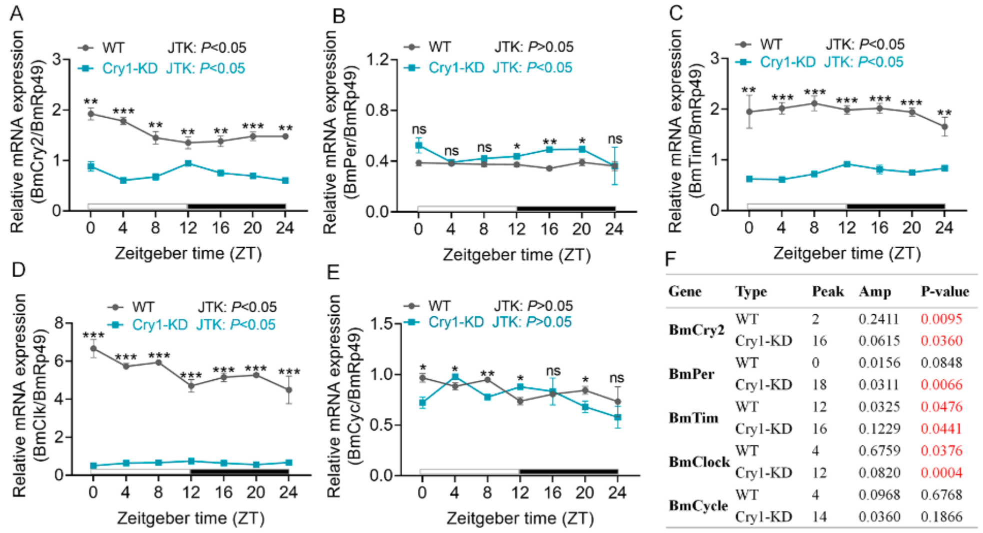

Gene	Type	Peak	Amp	P-value
BmCry2	WT	2	0.2411	0.0095
	Cry1-KD	16	0.0615	0.0360
BmPer	WT	0	0.0156	0.0848
	Cry1-KD	18	0.0311	0.0066
BmTim	WT	12	0.0325	0.0476
	Cry1-KD	16	0.1229	0.0441
BmClock	WT	4	0.6759	0.0376
	Cry1-KD	12	0.0820	0.0004
BmCycle	WT	4	0.0968	0.6768
	Cry1-KD	14	0.0360	0.1866

Figure S4. Effect of *BmCry1* gene knockout on clock gene expression. **(A-E)** Transcripts of the clock genes were analyzed with qRT-PCR using *BmRp49* as the internal control ($n = 3$). Cry1-KD and WT cells were cultured at 26 ° C in LD 12:12. Samples were analyzed at 4 h intervals for 24 h. **(F)** The expression rhythm of the clock genes was analyzed by JTK_CYCLE. JTK: $p < 0.05$ indicated that the gene expression had a 24-hour circadian rhythm, and JTK: $p > 0.05$ indicated no 24-h circadian rhythm in gene expression. The white lines indicate photophase

and the black lines indicate scotophase. WT, wild-type cells; Cry1-KD, *BmCry1* knockdown cells. *, $P \le 0.05$; **, $P \le 0.01$; ***, $P \le 0.001$; ns, non-significant.

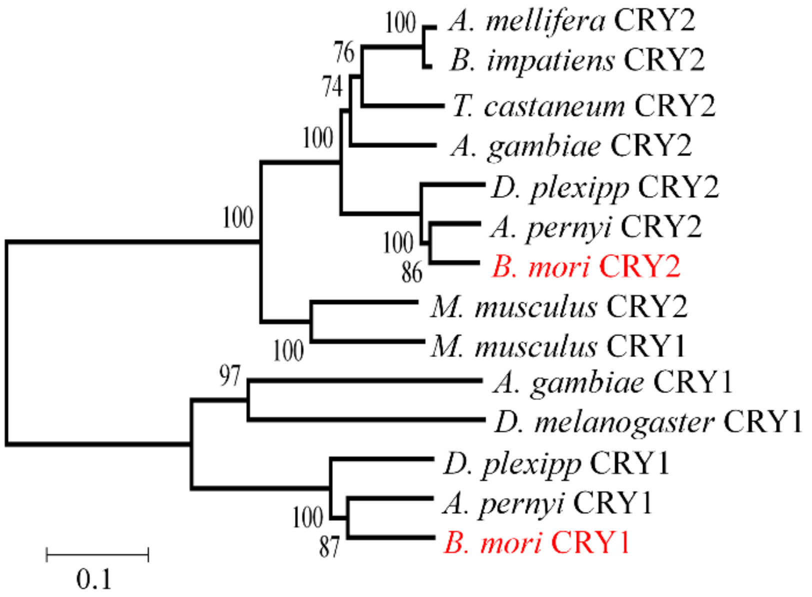

Figure S5. Molecular evolution and phylogenetic tree of CRY1 and CRY2 in insects. Two forms of CRY exist in insects, CRY1 and CRY2. The former is further from vertebrate *M.musulus* CRY in term of evolutionary distance, while the latter shows high homology with *M.musulus* CRYs. *A. mellifera, B. impatiens* (both Hymenoptera) and *T.castaneum* (Coleoptera) express CRY2 exclusivley; *D. melanogaster* expresses CRY1, exclusively; and *B. mori*, *A. pernyi* and *D.plexipp*, (all Lepidoptera) express both CRY1 and CRY2.

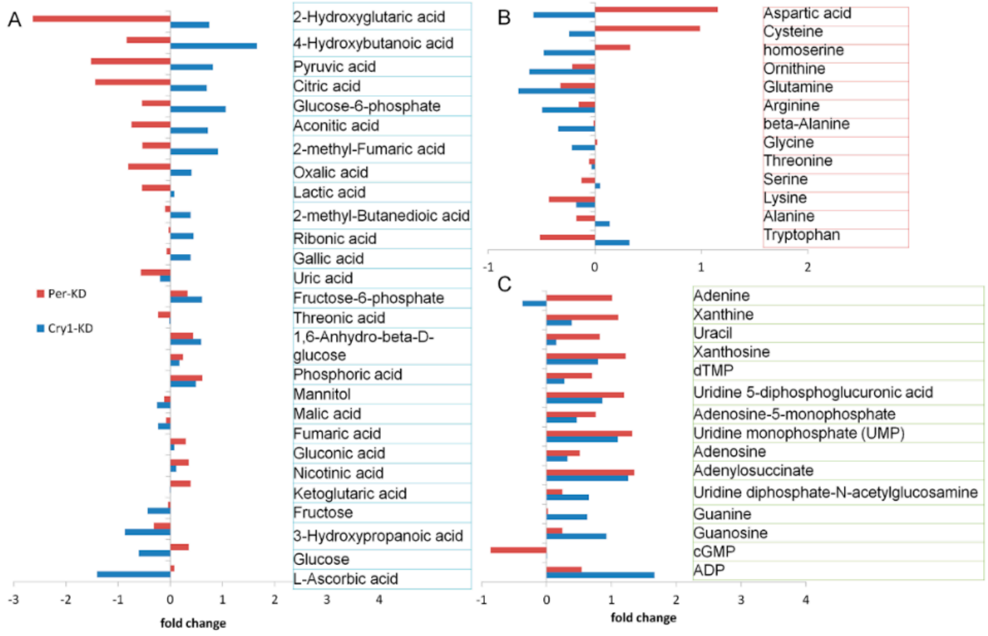

Figure S6. Comparison of metabolites in Cry1-KD and Per-KD cell lines. **(A)** Changes in sugars and organic acids involved in glucose and lipid metabolism compared with WT cells; **(B)** changes in amino acids compared with WT cells; **(C)** changes in nucleotides compared with WT cells. WT, wild-type BmN cells; Cry1-KD, *BmCry1* knockdown BmN cells; Per-KD, *BmPer* knockdown BmN cells; Log2 (fold change), Logarithm of Cry1-KD/WT or Per-KD/WT

fold change (fold change > 0 means Cry1-KD or Per-KD > WT; fold change < 0 means Cry1-KD or Per-KD < WT).

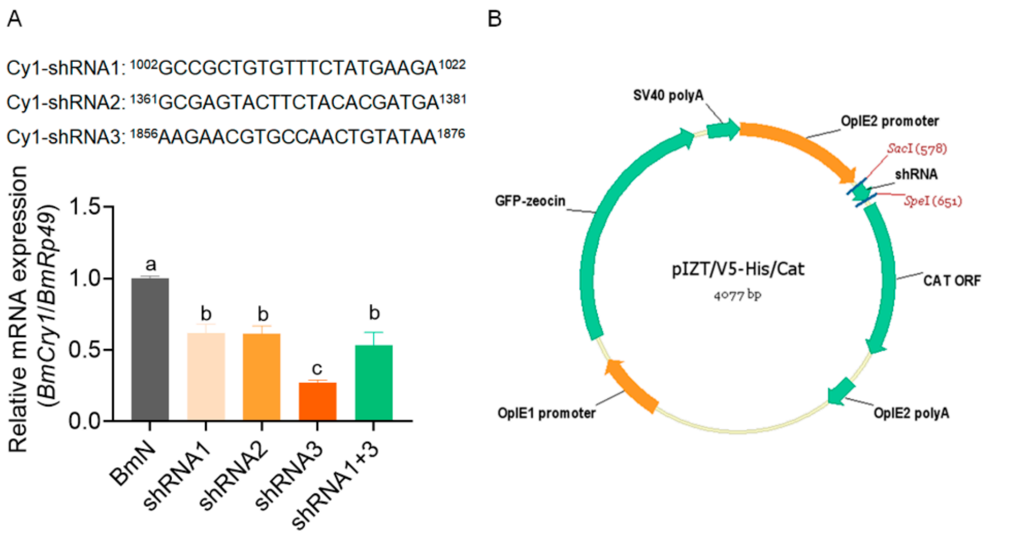

Figure S7. Knockdown efficiency of *BmCry1* and interference vector. **(A)** Three interference target sites were designed at 1002-1022, 1361-1381 and 1856-1876 of *BmCry1* mRNA, respectively. *BmCry1* mRNA levels were detected by qRT-PCR after transfection. **(B)** pIZT/V5-His/Cat vector with green fluorescent labeling for interference.

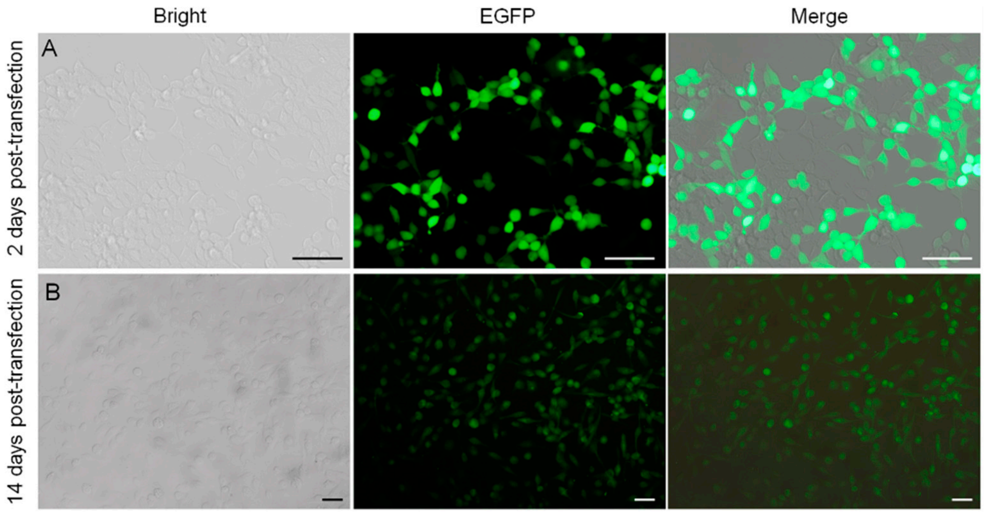

Figure S8. Transfection efficiency of shRNA-Cry1 expression vector transfected to BmN cells. **(A)** The number of green-labeled cells after 2 days of transfection. **(B)** The number of green-labeled cells after 14 days of transfection. bar = 200 μm.

Table S1. Primers used in this study

Primer name	Sequence
Ribosomal protein 49 (BmRp49)	F: CAGGCGGTTCAAGGGTCAATAC<br>R: TGCTGGGCTCTTTCCACGA
Hexokinase (BmHk)	F: ATGCTATGCGGCTCGAC<br>R: TGTACCACCCAAATCCAA
6-Phosphofructokinase (Bm6Pfk)	F: CTTCATTGGCTCGTTGAG<br>R: CATTGCTGTTTTGCTATTCT

Pyruvate kinase (BmPk)	F: CCATCGCATTGGACACTA<br>R: CCTGGCTTCACGACATTC
Cryptochrome 1 (BmCry1)	F: CCACGACAATCCGTCTCTT<br>R: GGTTGTACCCGACCACTTTCG
Cryptochrome 2 (BmCry2)	F: TTGTCCGTGAAGAGAGAGCG<br>R: AGAGAGAAAAGCCTGGGTGG
Period (BmPer)	F: GAAACGGAAACTGTATCGC<br>R: GAGGCAACAGAAGTAGTCA
Timeless (BmTim)	F: CTCTGCTCGGTCTTGTCATT<br>R: TGCACGGCTTGAGACCATTA
Clock (BmClk)	F: TGAACTCACATCCTGCTACT<br>R: CTTTCTTGCTTGGCGTTT
Cycle (BmCyc)	F: GCCCTGTCCCACTTACCT<br>R: TGGCTCCACCTCCTCCTT

Table S2. JTK_CYCLE analysis of circadian clock genes

Gene	Type	Peak (CT)	Amplitude	P-value
Cry2	WT	14	0.153	7.888×10^{-5}
	Cry1-KD	12	0.027	0.026
Per	WT	14	0.245	5.925×10^{-10}
	Cry1-KD	8	0.073	0.054
Tim	WT	14	0.039	0.012
	Cry1-KD	8	0.016	0.029
Clock	WT	12	0.019	0.008
	Cry1-KD	6	0.028	1
Cycle	WT	10	0.073	0.002
	Cry1-KD	14	0.014	0.344

Table S3. A list of differential metabolites obtained with GC-MS assay

**Metabolite**	**VIP**	**p value**	**Q value**	**Log_2(FC)**
4-Hydroxybutanoic acid	1.53	1.1E-10	1.0E-08	1.66
Inositol-1-phosphate	1.31	5.8E-05	4.4E-03	1.35
Glucose-6-phosphate	1.11	2.1E-03	1.2E-01	1.06
Guanosine	1.46	1.6E-07	1.4E-05	0.92
2-methyl-Fumaric acid	1.10	2.3E-03	1.3E-01	0.91
Pyruvic acid	1.53	6.6E-11	6.2E-09	0.82
2-Hydroxyglutaric acid	1.52	2.8E-10	2.6E-08	0.74
Inosine	1.40	2.9E-06	2.4E-04	0.73
Aconitic acid	1.27	1.4E-04	1.0E-02	0.72
Citric acid	1.39	4.6E-06	3.8E-04	0.70
Cholesterol	1.08	3.2E-03	1.7E-01	0.65
Glucose-6-phosphate	1.21	4.5E-04	3.0E-02	0.61
1,6-Anhydro-beta-D-glucose	1.42	1.3E-06	1.1E-04	0.59

Mannitol	1.48	2.5E-08	2.2E-06	0.49
Adenosine-5-monophosphate	1.01	6.9E-03	3.5E-01	0.47
Ribonic acid	1.49	7.8E-09	7.1E-07	0.44
Oxalic acid	1.51	1.2E-09	1.1E-07	0.40
Gallic acid	1.26	1.5E-04	1.1E-02	0.39
2-methyl-Butanedioic acid	1.05	4.6E-03	2.5E-01	0.39
Adenosine	1.18	7.0E-04	4.4E-02	0.33
Eicosanoic acid	1.19	5.7E-04	3.7E-02	0.33
Tryptophan	1.16	1.0E-03	6.2E-02	0.32
Uracil	1.08	2.9E-03	1.6E-01	0.15
Alanine	1.27	1.2E-04	8.7E-03	0.14
Lactic acid	1.05	4.5E-03	2.5E-01	0.07
Adenine	1.21	4.4E-04	3.0E-02	-0.17
Threitol	1.16	1.0E-03	6.1E-02	-0.17
Uric acid	1.04	5.1E-03	2.7E-01	-0.20
Glycine	1.30	7.3E-05	5.5E-03	-0.21
myo-inositol	1.17	8.4E-04	5.2E-02	-0.23
Fumaric acid	1.25	2.0E-04	1.4E-02	-0.24
Malic acid	1.47	7.2E-08	6.2E-06	-0.25
beta-Alanine	1.20	5.1E-04	3.4E-02	-0.34
Glycerol-2-phosphate	1.20	5.1E-04	3.4E-02	-0.35
Glucose	1.34	2.1E-05	1.7E-03	-0.43
homoserine	1.28	1.2E-04	8.5E-03	-0.48
Arginine	1.19	6.4E-04	4.1E-02	-0.50
Aspartic acid	1.30	7.1E-05	5.4E-03	-0.58
Glucose	1.31	5.1E-05	4.0E-03	-0.60
Ornithine	1.32	4.1E-05	3.3E-03	-0.62
Glyceric acid-3-phosphate	1.15	1.1E-03	6.7E-02	-0.70
Glutamine	1.37	7.8E-06	6.3E-04	-0.72
3-Hydroxypropanoic acid	1.53	1.1E-10	1.0E-08	1.66
Nicotinamide	1.31	5.8E-05	4.4E-03	1.35
Ethanolamine	1.11	2.1E-03	1.2E-01	1.06

VIP, PLS-DA first principal component variable importance projection value; p-value, *t-test* significance; q value, False-positive correction of t-test significance; Log_2(FC), logarithm of Cry1-KD/WT fold change.

Table. S4 A list of differentially expressed metabolites identified with LC-MS

**Metabolite**	**type**	**VIP**	**pcorr**	**p value**	**Log_2(FC)**
ADP	[M-H]-	4.7	0.9	0.006745	1.7
Adenylosuccinate	[M-H]-	10.6	0.9	0.029083	1.3
Flavin adenine dinucleotide (FAD)	[M+H]+	1.1	0.9	0.000016	1.2
Uridine monophosphate (UMP)	[M-H]-	7.7	0.9	0.000003	1.1

Flavine mononucleotide (FMN)	[M-H]-	1.2	0.9	0.011783	1.1
Uridine diphosphate-N-acetylglucosamine	[M-H]-	4.6	0.8	0.000323	0.7
Guanine	[M+H]+	1.4	0.9	0.000085	0.6
Glutathione	[M+H]+	7.2	0.8	0.000081	0.6
2-Chloroadenosine	[M+H]+	2.7	-0.8	0.000334	-1.1
Niacinamide	[M+H]+	3.3	-0.9	0.000000	-1.3
L-Ascorbic acid	[M+H]+	1.4	-0.9	0.000004	-1.4

Type, ionization mode, [M+H]+ positive ion mode, [M-H]- negative ion mode; VIP, PLS-DA first principal component variable importance projection value; p-value, *t-test* significance; pcorr, p (corr) value of s-plot correlation; Log_2(FC), logarithm of Cry1-KD/WT fold change.
